# The cost of exploitative leadership: how self-control depletion and organizational support shape employee outcomes

**DOI:** 10.3389/fpsyg.2025.1701529

**Published:** 2025-11-24

**Authors:** Xueqin Zhang, Zerui Wang, Xiyuan Jiang, Fei Xu

**Affiliations:** 1 Department of Business Administration, Hoseo University, Cheonan, Republic of Korea; 2 School of Marketing and International Business, Victoria University of Wellington, Wellington, New Zealand; 3 School of Digital Economy Industry, Guangzhou College of Commerce, Guangdong, China

**Keywords:** exploitative leadership, self-control depletion, turnover intention, workplace procrastination, perceived organizational support

## Abstract

**Introduction:**

Exploitative leadership, regarded as a manifestation of dark leadership, has recently attracted heightened academic interest because of its harmful consequences for employees. However, existing research has paid relatively little attention to the ways in which such leadership shapes critical employee outcomes, including intentions to leave and procrastination at work. Framed within conservation of resources theory, this study probes the latent pathways through which exploitative leadership erodes employee resources, precipitating both turnover intentions and procrastinatory conduct, while highlighting contextual levers that might temper these deleterious effects.

**Methods:**

To test the proposed hypotheses, we collected two-wave matched data from 296 full-time employees across various industries. Hierarchical regression and bootstrapping analyses were employed to assess the mediation and moderation effects.

**Results:**

The results unveiled that exploitative leadership precipitates heightened turnover intentions and procrastinatory behavior among employees, with self-control depletion operating as a pivotal mediator. Crucially, perceived organizational support functioned as a buffering mechanism, dampening both the impact of exploitative leadership on self-control depletion and its downstream indirect effects on turnover intention and workplace procrastination.

**Discussion:**

This study contributes to the growing literature on exploitative leadership and its consequences for employee attitudes and behaviors. It extends our understanding of the interactive effects between exploitative leadership and organizational support, and uncovers the critical role of self-control resources in shaping employees’ withdrawal responses. Practical implications for organizational leaders are also discussed.

## Introduction

Employee turnover continues to pose a pressing challenge for scholars and practitioners in human resource management (HRM) and organizational dynamics (Mengmeng et al., 2025; Veglio et al., 2025). Turnover not only signals workforce instability but also threatens organizational productivity and financial performance (Kizrak et al., 2024), thereby impeding sustainable development (Moffat et al., 2023). Consequently, attracting and retaining talent remains a critical priority for organizations (Kilson, 2025). At the same time, employee procrastination, recognized as a common behavioral pattern in the workplace (He et al., 2023), also generates a range of negative consequences for both employees and organizations (Kausar et al., 2025; Musteață and Holman, 2025). Thus, identifying HRM strategies aimed at reducing procrastination is also of great significance (Kang et al., 2023).

Leadership, as a key contextual factor in organizations, exerts a profound influence on employees’ attitudes and behaviors (Tejay and Winkfield, 2025; Zhang et al., 2025). Prior studies suggest that authentic and mindful leadership can effectively suppress employees’ turnover intention (Liu and Wong, 2023; Wibowo and Paramita, 2022), while authentic and humble leadership help reduce procrastination (Edú-Valsania et al., 2025; He et al., 2023). Nevertheless, prior research has largely focused on how positive leadership mitigates undesirable employee behaviors, whereas the harmful consequences of negative leadership approaches have received comparatively limited scholarly consideration. Among them, exploitative leadership stands out as a typical form of destructive leadership. Compared to positive leadership, negative leadership often exerts stronger and more pervasive effects on employees (Lyu et al., 2016; Wu et al., 2021). A core characteristic of exploitative leadership lies in leaders’ excessive self-interest, whereby they advance their own objectives while disregarding or even undermining the wellbeing of their subordinates (Schmid et al., 2019). Distinct from other detrimental leadership forms, exploitative leaders often project an illusory veneer of affability and warmth, thereby eluding easy detection (Schmid et al., 2019). This deceptive duality renders exploitative leadership distinct within the spectrum of negative leadership behaviors.

In today’s rapidly changing and uncertain organizational environments, retaining talent and minimizing detrimental workplace behaviors have become critical concerns (Raza et al., 2021; Shin and Grant, 2021). To achieve these goals, organizations must not only foster positive leadership practices but also actively prevent the damaging effects associated with negative leadership. Although the hazards of exploitative leadership have been recognized, its underlying mechanisms and boundary conditions remain insufficiently explored (Asim et al., 2024; Low et al., 2021). Anchored in conservation of resources (COR) theory (Hobfoll, 1989), the present research elucidates how self-control depletion functions as a mediator in the relationship between exploitative leadership and two employee outcomes—turnover intention and procrastination. Specifically, employees’ perception of exploitative leadership behaviors can deplete their self-control reserves, consequently making them more prone to consider leaving the organization or to engage in procrastination at work.

**Figure 1:**
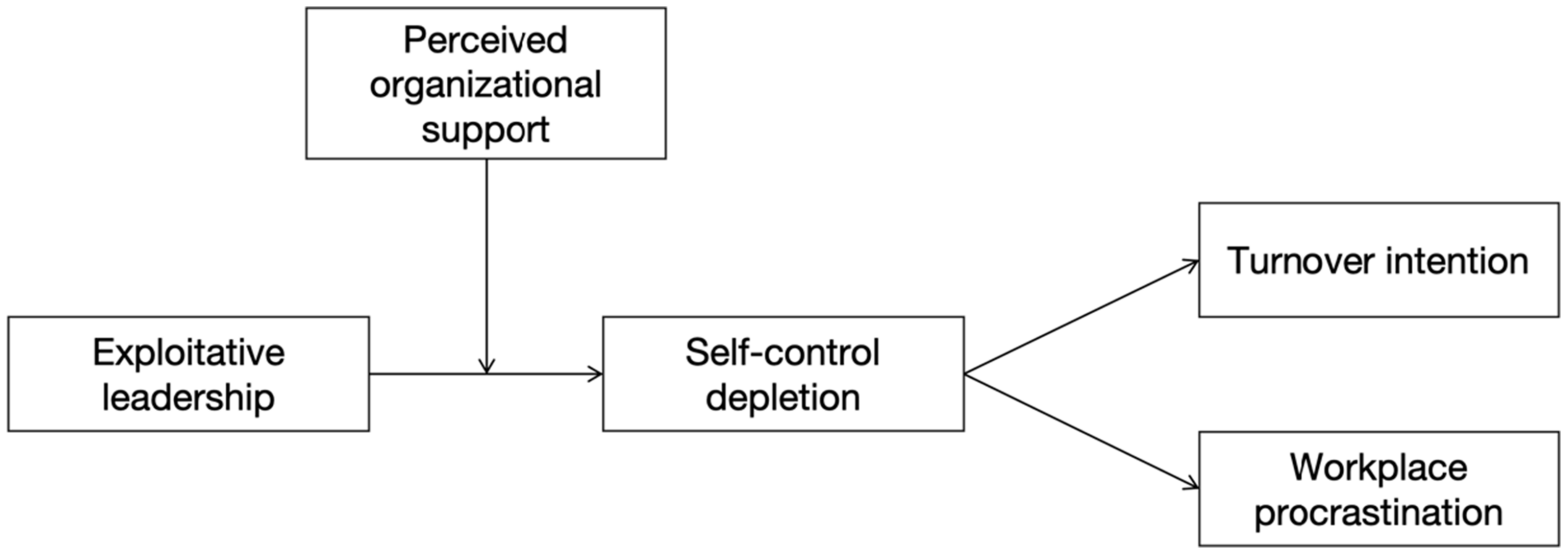
Proposed model.

Moreover, although prior research has explored the direct impact of exploitative leadership on outcomes such as turnover intention and procrastination (Majeed et al., 2023; Syed et al., 2021), relatively little emphasis has been placed on identifying potential moderating factors that might buffer these relationships. In particular, organizational-level resources that buffer the adverse impact of exploitative leadership have rarely been investigated. Evidence suggests that exploitative leadership cannot be entirely eliminated within organizations, yet its negative consequences may be alleviated through effective buffering mechanisms (Lee et al., 2018). COR theory suggests that when individuals obtain external resources in the face of resource depletion, the negative impact of loss can be alleviated (Chen et al., 2024; Wen et al., 2019). Building on this perspective, we introduce perceived organizational support (POS) as a potential moderator to examine its buffering role in the relationship between exploitative leadership and employee outcomes (see Figure 1). Prior studies indicate that POS not only enhances employee performance and coping capacity but also reduces negative work behaviors (Chen et al., 2009; McDaniel et al., 2021). Thus, we argue that high levels of organizational support may enable employees to better cope with the adverse cognitive states induced by exploitative leadership, thereby lowering turnover intention and procrastination.

In summary, this study makes several important contributions. First, it focuses on exploitative leadership, an emerging but underexplored form of negative leadership, thereby extending the understanding of destructive leadership and examining its effects on employees’ turnover intention and procrastination behavior. Previous research has primarily concentrated on how positive leadership mitigates undesirable employee behaviors, while the harmful effects of negative leadership have received comparatively little attention. Second, grounded in COR theory, this study incorporates self-control depletion as a mediating variable and POS as a moderating variable, constructing an integrative theoretical model that clarifies the mechanisms and boundary conditions of exploitative leadership. Notably, the role of organizational-level resources in buffering the negative impact of exploitative leadership has been rarely explored, and this study contributes to filling this gap. Third, by testing the model in the Chinese context, characterized by high power distance and collectivist cultural values (Farh et al., 2007; Medcof and Song, 2025), the study enriches cross-cultural leadership research and provides insights into why employees in such cultural settings may tolerate exploitative leadership. Methodologically, the study employs a two-wave longitudinal design involving employees from diverse industries, enhancing the robustness and temporal validity of the findings. Overall, this research not only advances theoretical understanding of the negative consequences of exploitative leadership but also offers practical guidance for managers seeking to mitigate adverse outcomes such as elevated turnover intentions and workplace procrastination.

## Literature review and hypothesis development

### Theoretical perspective

The COR theory (Hobfoll, 1989) has been extensively utilized in previous studies to account for how destructive leadership influences employees’ personal resources and, in turn, affects their behavioral outcomes (Asim et al., 2023; Shih et al., 2023). The present study, guided by COR theory, analyzes the pathways through which exploitative leadership contributes to employees’ turnover intention and procrastination, considering both direct effects and indirect effects mediated by self-control depletion. In addition, we further investigate the moderating role of POS in the relationship between exploitative leadership and self-control depletion.

In line with COR theory, the anticipation or experience of resource depletion compels individuals to adopt strategies aimed at conserving what remains while simultaneously attempting to accumulate additional reserves, such as material goods, time, and energy (Halbesleben et al., 2014; Hobfoll, 1989, 2001). In this sense, personal resources can either be depleted or accumulated. Exploitative leadership has been conceptualized as a damaging supervisory pattern that operates as a significant workplace strain, ultimately exhausting employees’ critical resources (Schmid et al., 2019). Employees rely on their personal resources to accomplish work tasks; however, exploitative leadership continuously drains these resources. Excessive loss of personal resources in employees often leads to increased psychological strain (Atingabili et al., 2025). Without timely recovery, such strain may hinder their performance, increase fatigue, and ultimately lead to higher turnover intention and greater procrastination.

COR theory highlights the pivotal role of resources in helping employees effectively cope with demanding work environments. The literature suggests that resource-rich individuals are comparatively better positioned to withstand stressors, while those with fewer resources face greater vulnerability (Halbesleben et al., 2009; Hobfoll et al., 2018). The supplementation of external resources can buffer the negative cognitive and emotional reactions triggered by perceived resource loss (Halbesleben et al., 2014; Hobfoll et al., 1990). Viewed through this lens, POS acts as an environmental safeguard, attenuating the erosive effect of exploitative leadership on employees’ depletion of self-regulatory capacity. POS has been recognized as an important resource that alleviates the negative consequences of demanding work environments (Qin et al., 2025). Employees who perceive higher organizational support tend to experience greater job satisfaction and lower turnover intentions, as documented in prior research (Kurtessis et al., 2017). By providing access to valuable resources, such support strengthens their ability to manage workplace demands effectively (Hobfoll et al., 2018). From this perspective, we propose that substantial organizational support constitutes a reinforcing resource mechanism that offsets the corrosive effects of exploitative leadership, whereas insufficient support magnifies employees’ vulnerability to its adverse consequences.

### Exploitative leadership, turnover intention and workplace procrastination

Previous research has extensively examined how to reduce employees’ turnover intention. Turnover intention is defined as the conscious decision or tendency of employees to leave their current job or organization (Pitts et al., 2011; Tett and Meyer, 1993). It is commonly regarded as a critical antecedent or proxy indicator of actual turnover (Ajzen, 1991, 2002; Bertelli, 2007; Sun and Wang, 2017; Tett and Meyer, 1993). Employees are more likely to develop turnover intention when they feel dissatisfied with their job or organization (Haque et al., 2019). As a negative response, turnover intention can potentially harm organizations over time, leading to knowledge loss, reduced performance, financial costs, and a decline in employee morale and productivity (Akgunduz and Eryilmaz, 2018; Kambur and Yildirim, 2023; Raza et al., 2021). Empirical studies have identified several key antecedents of turnover intention, including excessive workload, lack of career development opportunities, perceived justice, and leadership style (Chordiya, 2022; Hur and Abner, 2024).

Similar to turnover intention, procrastination also represents a form of negative response, conceptualized as a failure of self-regulation in which individuals deliberately postpone tasks or decisions despite being aware of the negative consequences (Steel, 2007). Workplace procrastination is widespread and tends to emerge more frequently when employees’ personal resources are depleted (Steel, 2007). Evidence shows that procrastination harms both personal and organizational outcomes by lowering individual performance, raising psychological stress (Koppenborg and Klingsieck, 2022; Sirois, 2014a, 2014b), reducing productivity (Özüdogru et al., 2024), and imposing economic costs (Nguyen et al., 2013). Its main triggers include monotonous or unengaging work environments and low levels of work motivation (Choy and Cheung, 2018; Özüdogru et al., 2024).

In organizational leadership research, exploitative leadership has been delineated by Schmid et al. (2019) as a destructive managerial pattern distinguished by self-serving, manipulative, and resource-eroding behaviors. Exploitative leadership is inherently detrimental to both individuals and organizations, as self-serving leaders covertly exploit and manipulate subordinates by imposing excessive workloads, demeaning and undermining them, and even obstructing their development (Schmid et al., 2019). Consequently, employees often perceive exploitative leadership as a stressor that depletes their personal resources. According to COR theory, under such circumstances, employees adopt defensive strategies to safeguard their remaining resources (Hobfoll, 2011). In cultural contexts characterized by high power distance, such as China, employees may be more inclined to tolerate and accept uncivil behaviors from higher-level leaders (Moon et al., 2018). Given the significant status gap, employees often find it difficult to directly resist exploitative leaders. Instead, they may respond in more covert and indirect ways, such as developing turnover intention or engaging in procrastination, to prevent further resource loss (Hofstede, 2001). Based on prior evidence, we propose the hypotheses:

*H1a.* Exploitative leadership increases employees’ turnover intention.

*H1b.* Exploitative leadership contributes to higher levels of workplace procrastination among employees.

### Self-control depletion as a mediator

From a COR-theoretical perspective, we extend that the influence of exploitative leadership on employees’ turnover likelihood and procrastination is transmitted through the depletion of self-control resources. Self-control depletion refers to a state in which individuals experience reduced cognitive resources and diminished willpower after prolonged self-regulation, representing a shortage of self-control resources (Bolton et al., 2012; Hagger et al., 2010). When employees lack such resources, they find it difficult to effectively regulate their thoughts, emotions, and behaviors, which in turn undermines task performance, effort, and self-regulation capacity.

We argue that when employees are persistently subjected to the pressure and extra demands imposed by exploitative leaders, they must consume self-control resources to suppress negative thoughts, emotions, or impulses toward the leader in order to meet expectations. This repeated self-regulation gradually depletes their limited resources (Baumeister et al., 2018; Joosten et al., 2014). COR theory posits that employees subjected to exploitative and manipulative behaviors by leaders tend to focus on protecting their remaining resources to avoid additional loss (Baumeister et al., 2007; Hagger et al., 2010). Consequently, employees tend to adopt defensive strategies such as attitudinal withdrawal (manifested as turnover intention) or behavioral withdrawal (manifested as workplace procrastination) to avoid further resource loss.

If such self-control depletion is not promptly restored, employees may struggle to recover from the exhausted state and sustain optimal performance. In this context, they often lack sufficient willpower, energy, and motivation to effectively cope with daily tasks. To regain psychological balance and alleviate stress, employees may develop turnover intention or exhibit procrastination behavior, such as reducing work engagement, mismanaging time, or even diverting work hours to personal matters (Sarwar et al., 2020). Based on the preceding discussion, we advance the following hypotheses:

*H2a.* Self-control depletion serves as a mediator in the relationship between exploitative leadership and employees’ turnover intention.

*H2b.* Self-control depletion serves as a mediator in the relationship between exploitative leadership and employees’ workplace procrastination.

### The moderating role of POS

As a vital work-related resource, POS provides significant value to employees. As a supportive and positive work condition, it encompasses not only material resources such as training and development opportunities and salary increases (Eisenberger et al., 1986; Probst et al., 2020; Rhoades and Eisenberger, 2002), but also psychological resources, including fostering employees’ sense of belonging (Waller, 2021) and recognizing their contributions. POS is defined as “the perception concerning the extent to which the organization values employees’ contributions and cares about employees’ wellbeing” (Eisenberger et al., 1986). High levels of POS help employees cope with stress and maintain positive attitudes (Côte et al., 2021), while fulfilling their socio-emotional needs for care, respect, and recognition (Wang and Xu, 2019). Low POS, in contrast, may trigger counterproductive work behaviors and reduce employees’ contributions beyond formal job requirements (Ike et al., 2024).

Drawing on COR theory, individuals utilize resources to handle difficult environments, seeking to acquire and conserve them. The provision of external resources offsets depletion, thereby reducing both stress and dissatisfaction (Wen et al., 2019). Organizational contexts, including the support provided by the organization, shape employees’ work-related responses (Al-Taie and Khattak, 2024; Kurtessis et al., 2017). Notably, previous research has demonstrated that POS, as a work resource, helps employees effectively cope with stress and regulate their behavioral responses (McDaniel et al., 2021). In this regard, POS can serve as a key moderating variable when examining employees’ behavioral responses under conditions of stress and resource depletion. Thus, we propose the following hypothesis:

*H3.* The positive impact of exploitative leadership on self-control depletion is contingent on POS, where greater POS reduces the strength of this relationship.

### Conditional mediation of self-control depletion by POS

Based on COR theory, this study views POS as a key resource for coping with resource depletion. It provides both material and emotional support, helping employees manage stress more effectively (Li et al., 2020; Shi et al., 2024). Thus, when facing pressure from exploitative leadership, employees who perceive higher organizational support are less likely to procrastinate or intend to leave. We advance, accordingly, a moderated mediation model. Specifically, the indirect effect of exploitative leadership on turnover intention and workplace procrastination via self-control depletion is moderated by POS. That is, the effect weakens when organizational support is high. Accordingly, we put forward the following hypotheses:

*H4a.* POS serves as a boundary condition in the indirect pathway from exploitative leadership to employee turnover intention through self-control depletion, reducing the strength of this positive relationship when POS is high.

*H4b.* POS serves as a boundary condition in the indirect pathway from exploitative leadership to employee workplace procrastination through self-control depletion, reducing the strength of this positive relationship when POS is high.

## Methods

### Sample and procedure

Full-time employees representing diverse sectors in China contributed survey responses via Wenjuanxing,^1^ a specialized online platform frequently leveraged for scholarly data collection (Cao et al., 2025; Peng et al., 2025). We leveraged the platform’s sample pool to identify potential participants. A convenience sampling method was adopted to select individuals who met the inclusion criteria. Eligibility for the study was limited to those in full-time positions who expressed readiness to participate in a two-wave data collection process.

To address potential concerns of common method variance, data were gathered at two distinct survey waves separated by roughly 2 weeks. Before participation, an invitation statement was issued, outlining the aims and basic procedures of the project. Respondents were informed that their input would be used strictly for scholarly purposes and would not be linked to any inappropriate or unlawful activities. They were further assured that involvement was entirely voluntary and anonymous, with the right to withdraw at any point. Completing the questionnaire signified consent to participate. As compensation, those who finished both survey waves were provided with a reward of RMB 4.

At the first wave (T1), questionnaires were distributed to 500 employees, capturing demographic data, perceptions of exploitative leadership, and organizational support. We initially gathered 373 valid responses. At the second wave (T2), participants were invited again to report their levels of self-control depletion, turnover intention, and workplace procrastination, producing 309 usable responses. Using the automatically generated unique IDs from the wenjuanxing platform, we paired participants’ responses across both waves. Following the exclusion of cases with implausibly fast completion times or contradictory responses, the resulting matched sample comprised 296 valid participants.

Among the 296 valid respondents, 51.35% were male (*n =* 152) and 48.65% female (*n =* 144). Age-wise, 28.38% were under 25, 44.93% were 25–29, 20.95% were 30–34, 4.73% were 35–39, and 1.01% were above 40. Regarding education, most participants (84.12%) held a bachelor’s degree, 9.8% had an associate degree, 4.73% a master’s degree, 0.34% a doctoral degree, and 1.01% had finished high school or technical secondary school. For organizational tenure, 51.01% had been with their organization for less than 3 years, 38.85% for 3–6 years, 8.45% for 6–9 years, 0.68% for 9–12 years, and 1.01% for over 12 years.

### Variable measurement

All research instruments were first designed in English and then adapted for use in Chinese following Brislin’s (1986) translation and back-translation procedure. Except where noted, respondents assessed each statement on a five-point Likert scale, with 1 indicating strong disagreement and 5 indicating strong agreement.

*Exploitative leadership*: The Exploitative Leadership Scale, developed by Schmid et al. (2019), comprises 15 items designed to assess employees’ perceptions of leaders’ self-serving and manipulative behaviors. One representative item stated, “My leader takes it for granted that my work can be used for his or her personal benefit.” Excellent reliability of the scale was exhibited (Cronbach’s *α* = 0.96).

*Self-control depletion*: The Self-Control Depletion Scale, developed by Twenge et al. (2004), consists of 5 items designed to measure the extent to which individuals feel their self-control resources have been depleted. One example item reads, “I feel like my willpower is gone.” The scale showed satisfactory reliability (Cronbach’s α = 0.89).

*Turnover intention*: The Turnover Intention Scale, developed by Chen and Wang (2019), comprises 4 items designed to assess employees’ intentions to leave their current organization. A representative item is, “I often want to leave my present organization or industry.” Reliability was high (Cronbach’s α = 0.92).

*Workplace procrastination*: Six items make up the Workplace Procrastination Scale (Kuehnel et al., 2016), which captures the extent to which employees postpone or delay work-related tasks. One example item reads, “I needlessly delayed finishing jobs, even when they were important.” The scale showed satisfactory reliability (Cronbach’s α = 0.88).

*Perceived organizational support*: The Perceived Organizational Support Scale, developed by Eisenberger et al. (1986), consists of 8 items. A sample item reads, “My organization really cares about my wellbeing.” Reliability was strong (Cronbach’s α = 0.91).

*Control variables*: Gender, age, education level, and organizational tenure were accounted for as control variables, given that these demographic factors may influence key constructs such as exploitative leadership (Wang et al., 2024).

## Results

### Confirmatory factor analysis

Ahead of hypothesis testing, confirmatory factor analyses were executed in Mplus 8.3 to ascertain that the five primary constructs investigated in this study were theoretically distinct. These analyses were performed to systematically assess the discriminant validity of the study variables. The results indicated that the hypothesized five-factor model demonstrated the best overall model fit (*χ*^2^ = 1392.434, df = 550, *χ*^2^/df = 2.532, CFI = 0.905; TLI = 0.897; RMSEA = 0.072; SRMR = 0.049). These findings support the discriminant validity among the five latent variables, suggesting that they are empirically distinguishable and conceptually non-overlapping. This provides a solid measurement foundation for the subsequent structural model analyses and hypothesis testing.

### Common method bias analysis

To probe the potential impact of common method variance, we formulated a six-factor measurement framework embedding a latent common factor, which was then evaluated in comparison with the initial five-factor model. The comparison revealed that the model incorporating the latent factor produced fit indices closely aligned with the baseline model (*χ*^2^ = 1456.849, df = 549, *χ*^2^/df = 2.654, CFI = 0.898; TLI = 0.889; RMSEA = 0.075; SRMR = 0.068), with no statistically meaningful differences observed, suggesting that common method variance has a negligible effect on the results and confirming the dataset’s adequacy for further analyses.

### Descriptive and correlations

Table 1 presents the descriptive statistics of the study variables, including means, standard deviations, and correlations. Exploitative leadership was positively correlated with self-control depletion (*r* = 0.678, *p* < 0.01), turnover intention (*r* = 0.608, *p* < 0.01), and procrastination behavior (*r* = 0.472, *p* < 0.01). Significant positive correlations with turnover intention (*r* = 0.632, *p* < 0.01) and procrastination behavior (*r* = 0.592, *p* < 0.01) were observed for self-control depletion. These findings are consistent with our theoretical expectations and provide preliminary support for our hypotheses.

**Table 1:** Descriptive statistics.

Variable	M	SD	1	2	3	4	5	6	7	8	9
1. Gender	1.486	0.501	1								
2. Age	27.686	3.843	−0.096	1							
3. Tenure	3.868	2.289	−0.076	0.773**	1						
4. Educational level	2.936	0.443	−0.012	0.295**	0.063	1					
5. Exploitative leadership	2.63	1.068	0.015	−0.189**	−0.186**	−0.07	(0.801)				
6. Self-control depletion	2.544	1.122	0.009	−0.303**	−0.261**	−0.148*	0.678**	(0.818)			
7. Turnover intention	2.095	1.061	−0.041	−0.206**	−0.210**	−0.081	0.608**	0.632**	(0.854)		
8. Procrastination at work	1.833	0.793	0.019	−0.150**	−0.086	−0.151**	0.472**	0.592**	0.579**	(0.744)	
9. Perceived organizational support	3.704	0.989	−0.104	0.191**	0.215**	0.026	−0.783**	−0.626**	−0.608**	−0.452**	(0.833)

*p < 0.05, **p < 0.01, ***p < 0.001.

### Hypothesis testing

All proposed hypotheses were tested using SPSS 27 in conjunction with the PROCESS macro, following the methodology recommended by Hayes (2017). Specifically, through the application of hierarchical multiple regression alongside mediation, moderation, and moderated mediation procedures, the hypothesized effects were rigorously tested.

A series of regression analyses were Initially conducted, controlling for demographic variables such as age, gender, education level, and tenure. The findings are summarized in Table 2. As shown in Models 6 and 9, exploitative leadership was positively associated with employees’ turnover intention (B = 0.585, *p* < 0.001) and procrastination behavior (B = 0.346, *p* < 0.001), thereby supporting Hypotheses 1a and 1b.

**Table 2:** Results of hypothesis testing.

Variable	Self-control depletion				Turnover intention			Procrastination at work		
	M1	M2	M3	M4	M5	M6	M7	M8	M9	M10
Gender	−0.022	−0.026	−0.074	−0.077	−0.114	−0.118	−0.108	0.016	0.014	0.023
Age	−0.104	−0.081	−0.081	−0.086	−0.044	−0.024	0.007	−0.042	−0.030	−0.001
Tenure	−0.302**	−0.126	−0.099	−0.086	−0.256*	−0.104	−0.056	−0.051	0.039	0.084
Educational level	−0.286	−0.206	−0.227*	−0.217*	−0.140	−0.071	0.007	−0.244*	−0.204*	−0.129
Exploitative leadership		0.677***	0.484***	0.446***		0.585***	0.329***		0.346***	0.102*
Self-control depletion							0.378***			0.360***
Perceived organizational support			−0.272***	−0.429***						
Exploitative leadership ✕ Perceived organizational support				0.164**						
R^2^	0.090	0.487	0.508	0.523	0.051	0.381	0.463	0.030	0.237	0.370

*p < 0.05, **p < 0.01, ***p < 0.001.

Table 3 presents the results of the mediation analyses. Self-control depletion significantly mediated the relationship between exploitative leadership and turnover intention (indirect effect = 0.256, 95% CI [0.158, 0.366]), supporting Hypothesis 2a. As shown in Model 7 of Table 2, self-control depletion was positively associated with turnover intention (B = 0.378, *p* < 0.001), and the effect of exploitative leadership remained significant (B = 0.329, *p* < 0.001), indicating partial mediation. Similarly, self-control depletion also mediated the relationship between exploitative leadership and workplace procrastination (indirect effect = 0.244, 95% CI [0.230, 0.439]), supporting Hypothesis 2b. In Model 10, self-control depletion showed a significant positive association with workplace procrastination (B = 0.360, *p* < 0.001), while the effect of exploitative leadership remained significant (B = 0.102, *p* < 0.05), suggesting that self-control depletion partially mediated this relationship.

**Table 3:** Results of mediation and moderated mediation effect analyses.

Paths and effects	Estimates	95% CI	
		LLCI	ULCI
**Mediating effect**			
EL → SD → TI	0.256	0.158	0.366
EL → SD → PAW	0.244	0.230	0.439
**EL → SD → TI**			
Moderated mediation effect	0.062	0.021	0.125
High POS (+1SD)	0.230	0.136	0.347
Low POS (−1SD)	0.107	0.030	0.199
**EL → SD → PAW**			
Moderated mediation effect	0.059	0.020	0.112
High POS (+1SD)	0.219	0.136	0.325
Low POS (−1SD)	0.102	0.027	0.190

EL, exploitative leadership; SD, self-control depletion; TI, turnover intention; PAW, procrastination at work; POS, perceived organizational support.

**Figure 2:**
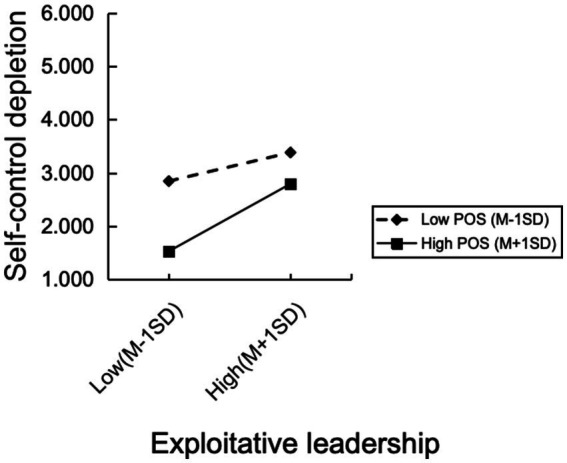
Moderating role of POS.

An interaction analysis was carried out to test the moderating effect of POS on the link between exploitative leadership and outcomes, while accounting for all control variables. Significant prediction of self-control depletion by the interaction term (B = 0.164, *p* < 0.01) is shown in Model 4 of Table 2, providing evidence in favor of Hypothesis 3. Figure 2 illustrates this interaction effect, indicating that the impact of exploitative leadership on self-control depletion was more pronounced when POS was high. Specifically, when POS was one standard deviation above the mean, exploitative leadership was positively associated with self-control depletion (simple slope = 0.592, *p* < 0.001). This association weakened when POS was one standard deviation below the mean (simple slope = 0.251, *p* < 0.01).

Finally, analyses for Hypotheses 4a and 4b were conducted via Model 7 of the PROCESS macro, utilizing bootstrapping to obtain empirically derived estimates of the effects. As shown in Table 3, the indirect effects of exploitative leadership on both turnover intention and workplace procrastination via self-control depletion were moderated by POS. Specifically, The extent to which self-control depletion mediated the relationship between exploitative leadership and turnover intention varied with POS, being considerably stronger under high support (indirect effect = 0.230, 95% CI [0.136, 0.347]) than under low support conditions (indirect effect = 0.107, 95% CI [0.030, 0.199]), thereby providing nuanced support for Hypothesis 4a. For workplace procrastination, a comparable pattern was detected: the mediating effect was more pronounced under elevated POS (indirect effect = 0.219, 95% CI [0.136, 0.325]) than under reduced support (indirect effect = 0.102, 95% CI [0.027, 0.190]), lending further support to Hypothesis 4b. Table 4 summarizes the results of all hypotheses tested.

**Table 4:** Summary of hypotheses testing results.

Hypothesis	Statement	Result	Supported
H1a	Exploitative leadership increases employees’ turnover intention.	B = 0.585, *p* < 0.001	Supported
H1b	Exploitative leadership contributes to higher levels of workplace procrastination among employees.	B = 0.346, *p* < 0.001	Supported
H2a	Self-control depletion serves as a mediator in the relationship between exploitative leadership and employees’ turnover intention.	indirect effect = 0.256, 95% CI [0.158, 0.366]	Supported
H2b	Self-control depletion serves as a mediator in the relationship between exploitative leadership and employees’ workplace procrastination.	indirect effect = 0.244, 95% CI [0.230, 0.439]	Supported
H3	The positive impact of exploitative leadership on self-control depletion is contingent on POS, where greater POS reduces the strength of this relationship.	B = 0.164, p < 0.01	Supported
H4a	POS serves as a boundary condition in the indirect pathway from exploitative leadership to employee turnover intention through self-control depletion, reducing the strength of this positive relationship when POS is high.	Estimate = 0.062, 95% CI [0.021, 0.125]	Supported
H4b	POS serves as a boundary condition in the indirect pathway from exploitative leadership to employee workplace procrastination through self-control depletion, reducing the strength of this positive relationship when POS is high.	Estimate = 0.059, 95% CI [0.020, 0.112]	Supported

## Discussion

This study explored the processes by which exploitative leadership affects employees’ turnover intentions and their tendency to engage in workplace procrastination. The results revealed that exploitative leadership increases self-control depletion, which in turn heightens both turnover intention and procrastination at work. However, this detrimental effect can be effectively mitigated by POS, particularly when such support is at a high level. These outcomes yield important contributions to theory and practical applications, discussed further in the subsequent sections.

### Theorical contributions

This study makes important contributions to both theoretical development and contextual application. First, using data from full-time employees across multiple industries in China, we tested the proposed research framework and examined the direct and indirect effects of exploitative leadership on employees’ turnover intention and workplace procrastination, as well as the mediating role of self-control depletion. The results indicate that exploitative leadership significantly increases turnover intention and procrastination, consistent with previous research showing that destructive leadership undermines employee attitudes and exacerbates negative behaviors (Schyns and Schilling, 2013). Specifically, the positive association between exploitative leadership and turnover intention supports prior findings that employees are more likely to disengage and seek alternative employment when facing unfair or self-serving leaders (Syed et al., 2021). Similarly, the link between exploitative leadership and procrastination extends existing research by demonstrating that negative leadership can deplete employees’ self-regulatory resources, which leads to behavioral withdrawal (Majeed et al., 2023).

Second, this study confirms the mediating role of self-control depletion in the relationship between exploitative leadership and employees’ turnover intention and procrastination, responding to previous calls for examining multiple pathways linking exploitative leadership to employee outcomes (Asim et al., 2024; Low et al., 2021). The findings indicate that self-control depletion is a key mechanism through which negative leadership shapes employees’ withdrawal-oriented attitudes and behaviors, consistent with prior research highlighting that adverse cognitive states are an important pathway for work-related stressors, especially exploitative leadership, to influence employees’ retreat behaviors (Nie and Wang, 2025).

Finally, the study highlights the moderating role of POS. Results show that for employees with high POS, the indirect effects of exploitative leadership on withdrawal-related attitudes and behaviors via self-control depletion are significantly weakened. This finding supports the proposition of COR theory that contextual resources can buffer processes of resource loss (Li et al., 2020; Côte et al., 2021). Moreover, the results indicate that POS functions as a critical organizational resource and serves as a stable predictor of constructive employee attitudes and behaviors, offering theoretical guidance for organizational management.

### Practical implications

Based on the findings of this study, exploitative leadership increases employees’ self-control resource depletion, which in turn elevates turnover intention and procrastination behavior. POS, however, can effectively mitigate these negative effects. Accordingly, the following managerial recommendations are proposed.

First, organizations should actively prevent and reduce exploitative leadership behaviors. In the processes of leader selection and promotion, employing personality inventories or behavioral evaluations can help detect and exclude candidates exhibiting exploitative tendencies or other unfavorable behaviors (Guo et al., 2024; Wang et al., 2021). In addition, targeted leadership development programs should be implemented to enhance leaders’ emotional intelligence, empathy, and fairness in management. Organizations should implement confidential and secure feedback channels, enabling employees to report instances of inappropriate leadership promptly and safely (Asim et al., 2024).

Second, organizations should take measures to alleviate employees’ self-control resource depletion. This may include providing psychological support and counseling, such as stress management training and mental health services, to assist employees in replenishing and sustaining their self-regulatory resources. Furthermore, employees should be encouraged to engage with human resource personnel to discuss exploitative leadership issues and share coping strategies, thereby enhancing their ability to manage work-related stress (Asim et al., 2025).

Third, organizations should actively enhance employees’ perceptions of organizational support. High levels of perceived support can effectively buffer the negative impact of exploitative leadership on employees’ self-control resource depletion, thereby reducing turnover intention and procrastination. Specific measures include providing both material and psychological support (e.g., appropriate rewards, benefits, career development, and training opportunities), fostering a caring management culture, monitoring employees’ work-related stress and needs, and encouraging positive interactions between teams, leaders, and coworkers to strengthen employees’ sense of organizational care and belonging.

### Limitations and future directions

Despite offering valuable theoretical and practical insights, this study is not without limitations. First, although this study included full-time employees from various industries, it was conducted within the Chinese context, which is characterized by high power distance (Farh et al., 2007). This cultural factor may have amplified employees’ perceptions of exploitative leadership and their behavioral responses (Kong et al., 2025; Zhu et al., 2025), potentially limiting the generalizability of the findings. In low-power-distance cultures, employees may be more likely to resist or counteract exploitative behaviors (Sun et al., 2023). Future research should examine whether these relationships hold in such cultural contexts to clarify the cultural boundary conditions of exploitative leadership and enhance the external validity of the findings.

Second, given that all data were derived exclusively from employee self-reports, the possibility of common method variance cannot be ruled out. Personal perceptions may have shaped the evaluation of variables (Podsakoff et al., 2012). However, the use of a two-wave time-lagged design and the latent common factor method helped to mitigate this issue to some extent. To address this concern more effectively, future studies should consider collecting data from multiple sources, such as having managers and colleagues evaluate employees’ procrastination behavior.

Third, this study focused exclusively on self-control depletion as the mediating mechanism linking exploitative leadership to employees’ attitudes and behaviors. To gain a more comprehensive understanding, future research should examine additional mediators, such as perceived organizational injustice (Hodson et al., 1994) or negative emotions like anger. In addition, while our theoretical framework incorporates POS as a moderating factor, other variables may also influence employees’ reactions to exploitative leadership. Future studies could examine factors such as leader identification (Marstand et al., 2018) and additional boundary conditions to better understand the range of coping strategies that employees employ when facing exploitative leaders.

Finally, although this study primarily draws on COR theory, alternative theoretical perspectives can also help explain the proposed model. For example, social exchange theory emphasizes the norms of reciprocity in leader–employee relationships (Blau, 2017; Cropanzano and Mitchell, 2005). When employees perceive that they are being exploited by their leaders, these reciprocity norms are violated, which may trigger negative responses. In addition, an ethical leadership framework (Brown et al., 2005) offers insights into employees’ reactions to exploitative leadership from a moral and ethical perspective. While COR theory serves as the core analytical framework of this research, future studies could integrate these complementary perspectives to further strengthen the model’s generalizability and explanatory robustness.

## Conclusion

This study, based on COR theory, developed and tested a moderated mediation model. The results indicate that employees’ perceptions of exploitative leadership increase turnover intention and workplace procrastination through self-control depletion, while POS can buffer these negative effects. The study deepens understanding of the mechanisms and boundary conditions of exploitative leadership and extends the application of COR theory to negative leadership contexts. However, the research was conducted only in the Chinese high-power-distance cultural context, relied primarily on self-reported data, and focused solely on self-control depletion as a mediator and POS as a moderator. Future research could test the model in different cultural settings, collect multi-source data, and explore additional mediators and moderators to more comprehensively understand how employees cope with exploitative leadership.

## Data Availability

The datasets presented in this study can be found in online repositories. The names of the repository/repositories and accession number(s) can be found in the article/supplementary material.

## Footnotes

^1^ https://www.wjx.cn

## References

[ref1] Ajzen I. (1991). The theory of planned behavior. Organ. Behav. Hum. Decis. Process. 50, 179–211. doi: 10.1016/0749-5978(91)90020-T

[ref2] Ajzen I. (2002). Perceived behavioral control, self-efficacy, locus of control, and the theory of planned behavior. J. Appl. Soc. Psychol. 32, 665–683. doi: 10.1111/j.1559-1816.2002.tb00236.x

[ref3] Akgunduz Y. Eryilmaz G. (2018). Does turnover intention mediate the effects of job insecurity and co-worker support on social loafing? Int. J. Hosp. Manag. 68, 41–49. doi: 10.1016/j.ijhm.2017.09.010

[ref4] Al-Taie M. Khattak M. N. (2024). The impact of perceived organizational support and human resources practices on innovative work behavior: does gender matter? Front. Psychol. 15:1401916. doi: 10.3389/fpsyg.2024.1401916, PMID: 39006546 PMC11240868

[ref5] Asim M. Hashmi U. F. Nadeem M. A. Ghani U. Yi X. (2025). How exploitative leadership influences employees' negligence behavior? Int. J. Confl. Manag. 36, 823–840. doi: 10.1108/ijcma-12-2024-0320

[ref6] Asim M. Liu Z. Y. Ghani U. Nadeem M. A. Yi X. (2024). Abusive supervision and helping behavior among nursing staff: a moderated mediation model. J. Health Organ. Manag. 38, 724–740. doi: 10.1108/jhom-12-2023-0372 39008095

[ref7] Asim M. Liu Z. Y. Nadeem M. A. Ghani U. Khalid J. Xu Y. (2023). Relationship of abusive supervision and employees' helping behaviors: moderated-mediation perspective. Int. J. Confl. Manag. 34, 367–391. doi: 10.1108/ijcma-11-2021-0185

[ref8] Atingabili S. Chen H. Arboh F. Mensah I. A. Kewou N. Y. N. Maalisuo B. S. (2025). Exposure to workplace bullying and nurses’ turnover intentions nexus: a moderation-mediation analysis. BMC Psychol 13:671. doi: 10.1186/s40359-025-03008-0, PMID: 40598632 PMC12220608

[ref9] Baumeister R. F. Bratslavsky E. Muraven M. Tice D. M. (2018). “Ego depletion: is the active self a limited resource?” in Self-regulation and self-control. ed. Baumeister R. (London: Routledge), 16–44.

[ref10] Baumeister R. F. Vohs K. D. Tice D. M. (2007). The strength model of self-control. Curr. Dir. Psychol. Sci. 16, 351–355. doi: 10.1111/j.1467-8721.2007.00534.x

[ref11] Bertelli A. M. (2007). Determinants of bureaucratic turnover intention: evidence from the department of the treasury. J. Public Adm. Res. Theory 17, 235–258. doi: 10.1093/jopart/mul003

[ref12] Blau P. (2017). Exchange and power in social life. London: Routledge.

[ref13] Bolton L. R. Harvey R. D. Grawitch M. J. Barber L. K. (2012). Counterproductive work Behaviours in response to emotional exhaustion: a moderated mediational approach. Stress. Health 28, 222–233. doi: 10.1002/smi.1425, PMID: 22281803

[ref14] Brislin R. W. (1986). The wording and translation of research instruments. London: Routledge.

[ref15] Brown M. E. Treviño L. K. Harrison D. A. (2005). Ethical leadership: a social learning perspective for construct development and testing. Organ. Behav. Hum. Decis. Process. 97, 117–134. doi: 10.1016/j.obhdp.2005.03.002

[ref16] Cao Y. Li H. Jiang T. Dang J. Hou Y. (2025). Laughing off cyber spoofing: the role of self-deprecating humor in enhancing celebrities' interpersonal likeability. BMC Psychol. 13:501. doi: 10.1186/s40359-025-02841-7, PMID: 40361205 PMC12070663

[ref17] Chen Z. X. Eisenberger R. Johnson K. M. Sucharski I. L. Aselage J. (2009). Perceived organizational support and extra-role performance: which leads to which? J. Soc. Psychol. 149, 119–124. doi: 10.3200/socp.149.1.119-124, PMID: 19245052

[ref18] Chen H.-T. Wang C.-H. (2019). Incivility, satisfaction and turnover intention of tourist hotel chefs: moderating effects of emotional intelligence [article]. Int. J. Contemp. Hospit. Manag. 31, 2034–2053. doi: 10.1108/ijchm-02-2018-0164

[ref19] Chen G. Wang J. Huang Q. Sang L. Yan J. Chen R. . (2024). Social support, psychological capital, multidimensional job burnout, and turnover intention of primary medical staff: a path analysis drawing on conservation of resources theory. Hum. Resour. Health 22:42. doi: 10.1186/s12960-024-00915-y, PMID: 38898452 PMC11186187

[ref20] Chordiya R. (2022). A study of interracial differences in turnover intentions: the mitigating role of pro-diversity and justice-oriented management. Public Personnel Manage. 51, 235–260. doi: 10.1177/00910260211061824

[ref21] Choy E. E. H. Cheung H. (2018). Time perspective, control, and affect mediate the relation between regulatory mode and procrastination. PLoS One 13:e0207912. doi: 10.1371/journal.pone.0207912, PMID: 30532229 PMC6287844

[ref22] Côte K. Lauzier M. Stinglhamber F. (2021). The relationship between presenteeism and job satisfaction: a mediated moderation model using work engagement and perceived organizational support. Eur. Manag. J. 39, 270–278. doi: 10.1016/j.emj.2020.09.001

[ref23] Cropanzano R. Mitchell M. S. (2005). Social exchange theory: an interdisciplinary review. J. Manage. 31, 874–900. doi: 10.1177/0149206305279602

[ref24] Edú-Valsania S. Laguía A. Moriano J. A. (2025). Interpersonal dynamics of authentic leadership: effects on support perception and workplace procrastination. Psycholo Int 7:21. doi: 10.3390/psycholint7010021

[ref25] Eisenberger R. Huntington R. Hutchison S. Sowa D. (1986). Perceived organizational support. J. Appl. Psychol. 71, 500–507. doi: 10.1037/0021-9010.71.3.500

[ref26] Farh J.-L. Hackett R. D. Liang J. (2007). Individual-level cultural values as moderators of perceived organizational support–employee outcome relationships in China: comparing the effects of power distance and traditionality. Acad. Manag. J. 50, 715–729. doi: 10.5465/amj.2007.25530866

[ref27] Guo L. M. Luo J. L. Cheng K. (2024). Exploitative leadership and counterproductive work behavior: a discrete emotions approach. Pers. Rev. 53, 353–374. doi: 10.1108/pr-02-2021-0131

[ref28] Hagger M. S. Wood C. Stiff C. Chatzisarantis N. L. D. (2010). Ego depletion and the strength model of self-control: a Meta-analysis. Psychol. Bull. 136, 495–525. doi: 10.1037/a0019486, PMID: 20565167

[ref29] Halbesleben J. R. B. Harvey J. Bolino M. C. (2009). Too engaged? A conservation of resources view of the relationship between work engagement and work interference with family. J. Appl. Psychol. 94, 1452–1465. doi: 10.1037/a0017595, PMID: 19916655

[ref30] Halbesleben J. R. B. Neveu J. P. Paustian-Underdahl S. C. Westman M. (2014). Getting to the "COR": understanding the role of resources in conservation of resources theory. J. Manage. 40, 1334–1364. doi: 10.1177/0149206314527130

[ref31] Haque A. Fernando M. Caputi P. (2019). The relationship between responsible leadership and organisational commitment and the mediating effect of employee turnover intentions: an empirical study with Australian employees. J. Bus. Ethics 156, 759–774. doi: 10.1007/s10551-017-3575-6

[ref9001] Hayes A. F. (2017). Introduction to mediation, moderation, and conditional process analysis: A regression-based approach. Guilford Publications.

[ref32] He W. Zhang Z. Guo Q. (2023). More humility for leaders, less procrastination for employees: the roles of career calling and promotion focus. Leadersh. Organ. Dev. J. 44, 120–136. doi: 10.1108/lodj-03-2022-0140

[ref33] Hobfoll S. E. (1989). Conservation of resources: a new attempt at conceptualizing stress. Am. Psychol. 44, 513–524. doi: 10.1037//0003-066x.44.3.513, PMID: 2648906

[ref34] Hobfoll S. E. (2001). The influence of culture, community, and the nested-self in the stress process: advancing conservation of resources theory. Appl. Psychol. Int. Rev. Psychol. Appliquee Rev. Int. 50, 337–370. doi: 10.1111/1464-0597.00062

[ref35] Hobfoll S. E. (2011). Conservation of resource caravans and engaged settings. J. Occup. Organ. Psychol. 84, 116–122. doi: 10.1111/j.2044-8325.2010.02016.x

[ref36] Hobfoll S. E. Freedy J. Lane C. Geller P. (1990). Conservation of social resources: social support resource theory. J. Soc. Pers. Relat. 7, 465–478. doi: 10.1177/0265407590074004

[ref37] Hobfoll S. E. Halbesleben J. Neveu J. P. Westman M. (2018). Conservation of resources in the organizational context: the reality of resources and their consequences. Ann. Rev. Org. Psychol. Org. Behav. 5, 103–128. doi: 10.1146/annurev-orgpsych-032117-104640

[ref38] Hodson R. Creighton S. Jamison C. S. Rieble S. Welsh S. (1994). Loyalty to whom - workplace participation and the development of consent. Hum. Relat. 47, 895–909. doi: 10.1177/001872679404700802

[ref39] Hofstede G. (2001). Culture's recent consequences: using dimension scores in theory and research. Int. J. Cross Cult. Manag. 1, 11–17. doi: 10.1177/147059580111002

[ref40] Hur H. Abner G. (2024). What makes public employees want to leave their job? A meta-analysis of turnover intention predictors among public sector employees. Public Adm. Rev. 84, 115–142. doi: 10.1111/puar.13601

[ref41] Ike O. O. Eze I. C. Nnadozie E. E. (2024). Unlocking the mask: Perceived organizational support as a buffer of the inimical effect of organizational cynicism on organizational workplace deviance behaviour. Cham: Springer.

[ref42] Joosten A. van Dijke M. Van Hiel A. De Cremer D. (2014). Being "in control" may make you lose control: the role of self-regulation in unethical leadership behavior. J. Bus. Ethics 121, 1–14. doi: 10.1007/s10551-013-1686-2

[ref43] Kambur E. Yildirim T. (2023). From traditional to smart human resources management. Int. J. Manpow. 44, 422–452. doi: 10.1108/ijm-10-2021-0622

[ref44] Kang F. Li J. Y. Hua Y. Y. (2023). How and when does humble leadership enhance newcomer well-being. Pers. Rev. 52, 26–41. doi: 10.1108/pr-01-2021-0019

[ref45] Kausar F. Ijaz M. U. Rasheed M. Suhail A. Islam U. (2025). Empowered, accountable, and committed? Applying self-determination theory to examine work-place procrastination. BMC Psychol. 13:620. doi: 10.1186/s40359-025-02968-7, PMID: 40481579 PMC12144702

[ref46] Kilson G. A. (2025). Optimizing employee attraction and retention in hospitality and tourism: a systematic review of employer branding research. Adm. Sci. 15:153. doi: 10.3390/admsci15050153

[ref47] Kizrak M. Çinar E. Aydin E. Kemikkiran N. (2024). How psychological safety influences intention to leave? The mediation roles of networking ability and relational job crafting. Curr. Psychol. 43, 9485–9503. doi: 10.1007/s12144-023-05028-8

[ref48] Kong L. Ding H. Yu S. Wu L. (2025). Linking exploitative leadership and employees’ work–family conflict: the roles of employees’ power distance orientation and emotional exhaustion. J. Psychol. Afr. 34, 689–696. doi: 10.1080/14330237.2024.2425411

[ref49] Koppenborg M. Klingsieck K. B. (2022). Group work and student procrastination. Learn. Individ. Differ. 94:102117. doi: 10.1016/j.lindif.2022.102117

[ref50] Kuehnel J. Bledow R. Feuerhahn N. (2016). When do you procrastinate? Sleep quality and social sleep lag jointly predict self-regulatory failure at work. J. Organ. Behav. 37, 983–1002. doi: 10.1002/job.2084

[ref51] Kurtessis J. N. Eisenberger R. Ford M. T. Buffardi L. C. Stewart K. A. Adis C. S. (2017). Perceived organizational support: a meta-analytic evaluation of organizational support theory. J. Manage. 43, 1854–1884. doi: 10.1177/0149206315575554

[ref52] Lee S. Kim S. L. Yun S. (2018). A moderated mediation model of the relationship between abusive supervision and knowledge sharing. Leadersh. Q. 29, 403–413. doi: 10.1016/j.leaqua.2017.09.001

[ref53] Li X. Zhang Y. Yan D. Wen F. Zhang Y. (2020). Nurses' intention to stay: the impact of perceived organizational support, job control and job satisfaction [article]. J. Adv. Nurs. 76, 1141–1150. doi: 10.1111/jan.14305 31957044

[ref54] Liu Z. Y. Wong H. (2023). Linking authentic leadership and employee turnover intention: the influences of sense of calling and job satisfaction. Leadersh. Organ. Dev. J. 44, 585–608. doi: 10.1108/lodj-01-2023-0044

[ref55] Low Y. M. Sambasivan M. Ho J. A. (2021). Impact of abusive supervision on counterproductive work behaviors of nurses. Asia Pac. J. Hum. Resour. 59, 250–278. doi: 10.1111/1744-7941.12234

[ref56] Lyu Y. J. Zhou X. Li W. W. Wan J. B. Zhang J. Qiu C. H. (2016). The impact of abusive supervision on service employees' proactive customer service performance in the hotel industry. Int. J. Contemp. Hospit. Manag. 28, 1992–2012. doi: 10.1108/ijchm-03-2015-0128

[ref57] Majeed M. Fatima T. Irshad M. (2023). A wolf in sheep's clothing: the perils of exploitative leadership. Eur. J. Soc. Psychol. 53, 1216–1230. doi: 10.1002/ejsp.2970

[ref58] Marstand A. F. Epitropaki O. Martin R. (2018). Cross-lagged relations between perceived leader-employee value congruence and leader identification. J. Occup. Organ. Psychol. 91, 411–420. doi: 10.1111/joop.12192

[ref59] McDaniel B. T. O'Connor K. Drouin M. (2021). Work-related technoference at home and feelings of work spillover, overload, life satisfaction and job satisfaction. Int. J. Workplace Health Manag. 14, 526–541. doi: 10.1108/ijwhm-11-2020-0197

[ref60] Medcof J. Song L. (2025). Exploratory and exploitative leadership compared: evidence from China. Int. J. Comp. Manage. 12, 1–14.

[ref61] Mengmeng L. Abdul Aziz N. Omar M. K. (2025). Unveiling of key factors influencing turnover intention: a review of literature. Int. J. Acad. Res. Bus. Soc. Sci. 15:2. doi: 10.6007/IJARBSS/v15-i2/24609

[ref62] Moffat E. Rioux L. Scrima F. (2023). The relationship between environmental bullying and turnover intention and the mediating effects of secure workplace attachment and environmental satisfaction: implications for organizational sustainability. Sustainability 15:11905. doi: 10.3390/su151511905

[ref63] Moon C. Weick M. Uskul A. K. (2018). Cultural variation in individuals' responses to incivility by perpetrators of different rank: the mediating role of descriptive and injunctive norms. Eur. J. Soc. Psychol. 48, 472–489. doi: 10.1002/ejsp.2344

[ref64] Musteață I. Holman A. C. (2025). Factors of workplace procrastination: a systematic review. Soc. Sci. 14:380. doi: 10.3390/socsci14060380

[ref65] Nguyen B. Steel P. Ferrari J. R. (2013). Procrastination's impact in the workplace and the workplace's impact on procrastination. Int. J. Sel. Assess. 21, 388–399. doi: 10.1111/ijsa.12048

[ref66] Nie Q. Wang M. M. (2025). Exploitative leadership and employees' unethical behavior from the perspective of ego depletion theory: the moderating effect of microbreaks. J. Leadersh. Organ. Stud. 32, 120–131. doi: 10.1177/15480518241305683

[ref67] Özüdogru A. G. Görener A. Toker K. (2024). Effect of hospital employees' psychological capital on counterproductive work behavior: role of work alienation and procrastination. SAGE Open 14:21582440241271138. doi: 10.1177/21582440241271138

[ref68] Peng C. Zhang S. Wen F. Liu K. (2025). How loneliness leads to the conversational AI usage intention: the roles of anthropomorphic interface, Para-social interaction. Curr. Psychol. 44, 8177–8189. doi: 10.1007/s12144-024-06809-5

[ref69] Pitts D. Marvel J. Fernandez S. (2011). So hard to say goodbye? Turnover intention among U.S. federal employees. Public Adm. Rev. 71, 751–760. doi: 10.1111/j.1540-6210.2011.02414.x

[ref70] Podsakoff P. M. MacKenzie S. B. Podsakoff N. P. (2012). Sources of method bias in social science research and recommendations on how to control it. Annu. Rev. Psychol. 63, 539–569. doi: 10.1146/annurev-psych-120710-100452 21838546

[ref71] Probst T. M. Petitta L. Barbaranelli C. Austin C. (2020). Safety-related moral disengagement in response to job insecurity: counterintuitive effects of perceived organizational and supervisor support. J. Bus. Ethics 162, 343–358. doi: 10.1007/s10551-018-4002-3

[ref72] Qin K. Yu Z. Cai Q. Jiang N. Chung K. S. (2025). How workplace Telepressure fuels job burnout among educators: mediated by work-related rumination and moderated by perceived organizational support. Behav. Sci. 15:1109. doi: 10.3390/bs15081109, PMID: 40867466 PMC12383041

[ref73] Raza B. St-Onge S. Ali M. (2021). Consumer aggression and frontline employees' turnover intention: the role of job anxiety, organizational support, and obligation feeling. Int. J. Hosp. Manag. 97:103015. doi: 10.1016/j.ijhm.2021.103015, PMID: 41181831

[ref74] Rhoades L. Eisenberger R. (2002). Perceived organizational support: a review of the literature. J. Appl. Psychol. 87, 698–714. doi: 10.1037//0021-9010.87.4.698 12184574

[ref75] Sarwar A. Naseer S. Zhong J. Y. (2020). Effects of bullying on job insecurity and deviant behaviors in nurses: roles of resilience and support. J. Nurs. Manag. 28, 267–276. doi: 10.1111/jonm.12917, PMID: 31788904

[ref76] Schmid E. A. Verdorfer A. P. Peus C. (2019). Shedding light on leaders' self-interest: theory and measurement of exploitative leadership [article]. J. Manage. 45, 1401–1433. doi: 10.1177/0149206317707810

[ref77] Schyns B. Schilling J. (2013). How bad are the effects of bad leaders? A meta-analysis of destructive leadership and its outcomes. Leadersh. Q. 24, 138–158. doi: 10.1016/j.leaqua.2012.09.001

[ref78] Shi Y. Wang L. Zhang J. Zhao J. Peng J. Cui X. . (2024). The influence of effort-reward imbalance and perceived organizational support on perceived stress in Chinese nurses: a cross-sectional study. BMC Nurs. 23:701. doi: 10.1186/s12912-024-02327-8, PMID: 39343874 PMC11440884

[ref79] Shih F. C. Yeh S. C. J. Hsu W. L. (2023). Abusive supervision and employee well-being of nursing staff: mediating role of occupational stress. J. Adv. Nurs. 79, 664–675. doi: 10.1111/jan.15538, PMID: 36511427

[ref80] Shin J. Grant A. M. (2021). When putting work off pays off: the curvilinear relationship between procrastination and creativity. Acad. Manag. J. 64, 772–798. doi: 10.5465/amj.2018.1471

[ref81] Sirois F. M. (2014a). Out of sight, out of time? A meta-analytic investigation of procrastination and time perspective. Eur. J. Personal. 28, 511–520. doi: 10.1002/per.1947

[ref82] Sirois F. M. (2014b). Procrastination and stress: exploring the role of self-compassion. Self Identity 13, 128–145. doi: 10.1080/15298868.2013.763404

[ref83] Steel P. (2007). The nature of procrastination: a meta-analytic and theoretical review of quintessential self-regulatory failure. Psychol. Bull. 133, 65–94. doi: 10.1037/0033-2909.133.1.65, PMID: 17201571

[ref84] Sun R. S. Wang W. J. (2017). Transformational leadership, employee turnover intention, and actual voluntary turnover in public organizations. Public Manag. Rev. 19, 1124–1141. doi: 10.1080/14719037.2016.1257063

[ref85] Sun Z. Wu L.-Z. Ye Y. Kwan H. K. (2023). The impact of exploitative leadership on hospitality employees’ proactive customer service performance: a self-determination perspective. Int. J. Contemp. Hosp. Manag. 35, 46–63. doi: 10.1108/IJCHM-11-2021-1417

[ref86] Syed F. Naseer S. Akhtar M. W. Husnain M. Kashif M. (2021). Frogs in boiling water: a moderated-mediation model of exploitative leadership, fear of negative evaluation and knowledge hiding behaviors. J. Knowl. Manag. 25, 2067–2087. doi: 10.1108/jkm-11-2019-0611

[ref87] Tejay G. P. Winkfield M. (2025). Does leadership approach matter? Examining behavioral influences of leaders on employees’ information security compliance. Inf. Syst. Front. 22, 1–21. doi: 10.1007/s10796-025-10592-4

[ref88] Tett R. P. Meyer J. P. (1993). Job satisfaction, organizational commitment, turnover intention, and turnover: path analyses based on meta-analytic findings. Pers. Psychol. 46, 259–293. doi: 10.1111/j.1744-6570.1993.tb00874.x

[ref89] Twenge J. Muraven M. Tice D. (2004). Measuring state self-control: Reliability, validity, and correlations with physical and psychological stress. San Diego, CA, USA: San Diego State University.

[ref90] Veglio V. Romanello R. Pedersen T. (2025). Employee turnover in multinational corporations: a supervised machine learning approach. Rev. Manag. Sci. 19, 687–728. doi: 10.1007/s11846-024-00769-7

[ref91] Waller L. (2021). “Fostering a sense of belonging in the workplace: enhancing well-being and a positive and coherent sense of self” in The Palgrave handbook of workplace well-being. ed. Dhiman S. (Cham: Springer), 341–367.

[ref92] Wang Z. Ren S. Chadee D. Chen Y. (2024). Employee ethical silence under exploitative leadership: the roles of work meaningfulness and moral potency [article]. J. Bus. Ethics 190, 59–76. doi: 10.1007/s10551-023-05405-0

[ref93] Wang Z. N. Ren S. Chadee D. Sun C. W. (2021). The influence of exploitative leadership on hospitality employees' green innovative behavior: a moderated mediation model. Int. J. Hosp. Manag. 99:103058. doi: 10.1016/j.ijhm.2021.103058

[ref94] Wang Z. Xu H. Y. (2019). When and for whom ethical leadership is more effective in eliciting work meaningfulness and positive attitudes: the moderating roles of core self-evaluation and perceived organizational support. J. Bus. Ethics 156, 919–940. doi: 10.1007/s10551-017-3563-x

[ref96] Wen J. Huang S. S. Hou P. P. (2019). Emotional intelligence, emotional labor, perceived organizational support, and job satisfaction: a moderated mediation model. Int. J. Hosp. Manag. 81, 120–130. doi: 10.1016/j.ijhm.2019.01.009

[ref97] Wibowo A. Paramita W. (2022). Resilience and turnover intention: the role of mindful leadership, empathetic leadership, and self-regulation. J. Leadersh. Organ. Stud. 29:15480518211068735, 325–341. doi: 10.1177/15480518211068735

[ref98] Wu L. Z. Sun Z. Z. Ye Y. J. Kwan H. K. Yang M. Q. (2021). The impact of exploitative leadership on frontline hospitality employees' service performance: a social exchange perspective. Int. J. Hosp. Manag. 96:102954. doi: 10.1016/j.ijhm.2021.102954

[ref99] Zhang S. Liu C. Lai C. Zhao B. Zhang C. Zhang Y. . (2025). The power of leadership transforming nurses’ professional attitudes through the balance theory: a cross-sectional study. BMC Nurs. 24, 1–14. doi: 10.1186/s12912-025-03517-8, PMID: 40616151 PMC12231632

[ref100] Zhu L. Jin X. Kwak W. J. (2025). The relationship between exploitative leadership and counterproductive work behavior: the moderated mediation effect of power distance. SAGE Open 15:21582440251381247. doi: 10.1177/21582440251381247

